# Mutation analysis of *BRAF* and *KIT* in circulating melanoma cells at the single cell level

**DOI:** 10.1038/bjc.2012.12

**Published:** 2012-01-26

**Authors:** K Sakaizawa, Y Goto, Y Kiniwa, A Uchiyama, K Harada, S Shimada, T Saida, S Ferrone, M Takata, H Uhara, R Okuyama

**Affiliations:** 1 Department of Dermatology, Shinshu University School of Medicine, 3-1-1 Asahi, Matsumoto 390-8621, Japan; 2 Department of Dermatology, University of Yamanashi School of Medicine, 1110 Shimokawato, Chuo 409-3898, Japan; 3 Departments of Surgery, Immunology and Pathology, University of Pittsburgh Cancer Institute, 5117 Center Avenue Suite 2.26d, Pittsburgh, PA 15213, USA

**Keywords:** melanoma, circulating tumour cells, oncogene mutations, BRAF, KIT, HMW-MAA

## Abstract

**Background:**

The availability of molecular-targeted therapies for the treatment of melanoma has emphasised the need to identify mutations in target genes such as *BRAF* and *KIT*. Circulating tumour cells (CTC) are present in the peripheral blood of a significant proportion of cancer patients.

**Methods:**

High molecular weight melanoma-associated antigen (HMW-MAA) was used to isolate melanoma cells from peripheral blood as it is selectively expressed at high levels on melanomas. The HMW-MAA-positive cells were isolated using immunomagnetic beads. After removing CD45^+^ cells, CTC were identified by staining with MART-1- and gp100-specific antibodies (HMW-MAA^+^, CD45^−^, MART-1/gp100^+^). Single, isolated CTC were then subjected to *BRAF* and *KIT* mutational analysis.

**Results:**

CTC (HMW-MAA^+^, CD45^−^, MART-1/gp100^+^) were isolated from the blood of 11 patients and *BRAF* and *KIT* were sequenced in nine and four patients, respectively. The *BRAF* sequences identified in the CTC were inconsistent with those identified in autologous melanoma tumours in three patients and the *KIT* sequences were inconsistent in three patients. In addition, polyclonal *BRAF* mutations were identified in one patient and concomitant mutations in *BRAF* and *KIT* were identified in another patient.

**Conclusion:**

Melanoma cells show clonal heterogeneity. Therefore, CTC genotyping may be crucial for successful molecular-targeted therapy.

Therapies that target specific molecules markedly inhibit cancer growth in several malignancies, and provide valuable strategies for the treatment of advanced melanoma (Romano *et al*, 2011). In recent years, *BRAF* and *KIT* have become established therapeutic targets in melanoma patients showing activating mutations in these oncogenes. Significant clinical benefits have been achieved using the RAF kinase inhibitor, vemurafenib, to treat melanomas showing *BRAF* mutations and the receptor protein kinase inhibitor, imatinib, to treat melanomas showing *KIT* mutations (Flaherty *et al*, 2010; Carvajal *et al*, 2011; Chapman *et al*, 2011; Guo *et al*, 2011). Somatic missense activating mutations in *BRAF* have been identified in about 60% of primary melanoma lesions (Davies *et al*, 2002), and *KIT* mutations in 14% and 18% of acral lentiginous and mucosal melanomas, respectively (Woodman and Davies, 2010). However, it is crucial that genetic mutations present in the melanoma lesions are identified if we are to design tailor-made therapies for individual patients. The tumour genotypes that determine the selection of molecular-targeted therapies are usually identified in primary tumours; however, primary tumours are not always representative of metastases. For example, Terheyden *et al* (2010) reported the case of a melanoma patient with a primary lesion and lymph node metastases that showed a wild-type *KIT* genotype, but with lung metastasis harbouring a *KIT*^V559A^ mutation.

Circulating tumour cells (CTC) have been identified in peripheral blood from a significant proportion of patients with recurrent disease, and may still be detectable following removal of the primary tumour (Allard *et al*, 2004; Mocellin *et al*, 2006). It may be a source of valuable information because they can be obtained via routine blood sampling, they provide real-time information about a patient's current disease state, and their features may mirror those of recurrent tumours. However, CTC from melanoma patients have only been characterised to a limited extent. This limitation reflects, at least in part, difficulties in isolating these rare cells from the many circulating blood cells. Recently, melanoma cells were isolated from melanoma patients using immunomagnetic beads coated with antibodies specific for chondroitin sulphate proteoglycan (CSPG) 4 (Ulmer *et al*, 2004; Kitago *et al*, 2009; Suesskind *et al*, 2011). This tumour antigen, also known as high molecular weight melanoma-associated antigen (HMW-MAA), can be used to identify and isolate CTC from peripheral blood because it is expressed on the melanoma cell membrane in >85% of primary and metastatic melanoma lesions (Campoli *et al*, 2004). High affinity CSPG 4-specific antibodies are available, and the corresponding epitopes are not detectable on normal blood cells.

Therefore, the aim of the present study was to isolate intact CTC from peripheral blood using a three-step purification procedure incorporating positive selection with a pool of three HMW-MAA-specific monoclonal antibodies (mAbs) followed by negative selection with a CD45-specific mAb. The purity of the isolated CTC was monitored according to the expression of HMW-MAA and MART-1/gp100 (melanoma markers), and lack of CD45 expression (a haematopoietic cell marker). *BRAF* and *KIT* mutations in CTC were then examined at the single cell level and the genotypes compared with those of the primary and metastatic lesions.

## Materials and methods

### Melanoma cell lines, reagents, mAbs, and clinical specimens

Detailed procedures and patient information are described in Supplementary Information.

### Isolation of melanoma cells from peripheral blood

Heparinised blood samples (5 ml) were treated with RBC lysis solution (Qiagen, Germantown, MD, USA), after which >4 × 10^6^ mononuclear cells were obtained. Circulating tumour cells were isolated from the mononucleated cells using HMW-MAA-specific mAb-coated immunomagnetic beads (Dynabeads CELLection Pan Mouse IgG Kit; Invitrogen, Oslo, Norway) according to manufacturer's instructions with minor modifications. Melanoma cells were labelled with mAb before capture using immunomagnetic beads because previous work showed that this indirect technique is better than direct techniques for separating melanoma cells from the blood (Kitago *et al*, 2009). In brief, 6 *μ*l of a cocktail containing HMW-MAA-specific mAbs 763.74, VF1-TP41.2, and VT80.12 (final concentration of each mAb: 33 *μ*g ml^−1^) was added to 4 × 10^6^ cells suspended in 294 *μ*l phosphate-buffered saline (PBS) containing 0.1% bovine serum albumin (BSA). Samples were incubated with rotation at 22 r.p.m. at room temperature (Invitrogen) for 20 min. After washing with PBS/0.1% BSA, the samples were mixed with 25 *μ*l of immunomagnetic beads (anti-mouse IgG-coated), which included 1 × 10^7^ beads. The mixtures were incubated with rotation at 22 r.p.m. for an additional 30 min. Samples were then placed in a DYNAL MPC-S magnetic device (Invitrogen) for 2 min. Following removal of the fluid by careful pipetting and washing twice with PBS/0.1% BSA, cells were released from the beads by incubation with releasing buffer (Invitrogen) for 20 min on a mixing device. The mixture was then vigorously pipetted and replaced in the DYNAL MPC-S magnetic device for 2 min to isolate HMW-MAA^+^ cells. Human CD45-specific mAb-coated immunomagnetic beads (Invitrogen) were then used to remove contaminating white blood cells. After incubation and washing, the HMW-MAA^+^, CD45^−^ cell population was collected. The HMW-MAA^+^, CD45^−^ cells were then smeared onto a silane-coated glass slide (Dako, Carpinteria, CA, USA) and stained (EnVision System; Dako) with a cocktail of MART-1 and gp100 antibodies. The stained cells were then subjected to laser-capture microdissection using a PALM-MB microdissection system (PALM Microlaser Technologies, Bernried, Germany) and scored as positive when a clear signal was observed over at least 80% of the cell surface. MART-1/gp100^+^ (HMW-MAA^+^, CD45^−^, MART-1/gp100^+^) cells were then isolated individually and placed separately in adhesive tubes (Meiwafosis, Tokyo, Japan). These cells were referred to as CTC.

### DNA extraction

For extraction of genomic DNA from single CTC, 3 *μ*l of lysis buffer (10 mM Tris–HCl (pH 8.3), 50 mM KCl, 4 mg ml^−1^ proteinase K (Roche Diagnostics, Basel, Switzerland), and 3% Tween-20) was added to each tube. The tubes were subsequently vortexed. After centrifugation, each CTC was incubated for 16 h at 50°C, after which proteinase K was heat inactivated at 95°C for 10 min. As a negative control, empty tubes were incubated with lysis buffer. In addition, DNA was extracted from formalin-fixed, paraffin-embedded surgically resected tumour tissues. Three 6-*μ*m sections were cut from paraffin-embedded tissue blocks with a sterile microtome blade and mounted on glass slides. After deparaffinisation, tissues stained with methylene blue were dissected manually on an inverted microscope to select melanoma lesions. The dissected tissues were placed in sterile tubes and digested with a mixture containing 20 *μ*l of proteinase K and 180 *μ*l of 25 mM Tris–HCl at 37°C for 12 h. After completion of the incubation, proteinase K was heat inactivated at 80°C for 20 min.

### Analysis of *BRAF* and *KIT* mutations

Primers were designed to amplify exon 15 of *BRAF* and exons 11, 13, and 17 of *KIT*, all of which include mutational hot spots (Davies *et al*, 2002; Woodman and Davies, 2010). Exon 15 of *BRAF* was amplified using a hemi-nested PCR, and exons 11, 13, and 17 of *KIT* were amplified using nested PCR (Buttner *et al*, 1998; Hofmann *et al*, 2009; Lin *et al*, 2009). The primer sequences and PCR cycling conditions are shown in Supplementary Table S1. The first PCR amplification for exon 15 of *BRAF* was conducted in a 20-*μ*l reaction volume containing Ex Taq buffer with 2.0 mM MgCl_2_, 0.2 mM dNTP mixture, 0.3 *μ*M primers, 0.5 U of Ex Taq (Takara, Shiga, Japan), and 2 *μ*l of DNA template. Then, 0.2 *μ*l of the first PCR amplification product was used as template for the second PCR amplification, which was conducted in a 20-*μ*l reaction volume. *KIT* exons were amplified in singleplex reactions. Nested PCR for *KIT* exons, both the first and second PCR amplifications, was conducted in 20 *μ*l reaction volumes including 2 *μ*l of DNA template. The PCR amplification was performed in iCycler (Bio-Rad Laboratories, Hercules, CA, USA). After confirming the size of the PCR products on agarose gels, the products were purified using the QIAquick PCR Purification Kit (Qiagen). Direct sequencing was performed to identify mutations within exon 15 of *BRAF* and exons 11, 13, and 17 of *KIT*. The primer used for the forward reading reaction of *BRAF* exon 15 was 5′-TCATAATGCTTGCTCTGATAGGA-3′. The primers used for the forward reading reaction of *KIT* exons 11, 13, and 17 were the same as the forward inner primers used for nested PCR. Each sequencing reaction was carried out using the BigDye Terminator v3.1 cycle sequencing kit (Applied Biosystems, Foster City, CA, USA) in a 20-*μ*l volume containing purified PCR product and sequence primer. The temperature for the sequencing reaction was 96°C for 1 min, which was followed by 25 cycles at 96°C for 10 s, 54°C for 5 s, and 60°C for 4 min. The reaction products were precipitated with 95% ethanol and 3 mM sodium acetate, washed with 70% ethanol, resuspended in 20 *μ*l Hi-Di formamide and loaded onto an ABI PRISM 3100 Genetic Analyzer (Applied Biosystems). The analysis was conducted following the accepted PCR guidelines (Bustin *et al*, 2009).

## Results

### Melanoma cell separation in an *in-vitro* model experiment

**Figure 1:**
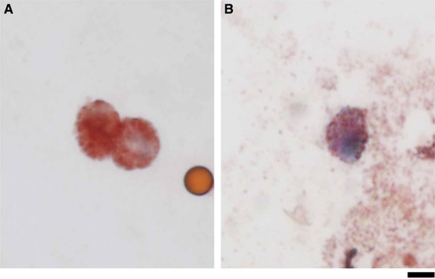
Melanoma cells isolated from peripheral blood. HMW-MAA^+^, CD45^−^ cells were isolated on immunomagnetic beads and stained with a mixture of MART-1 and gp100 antibodies. MART-1/gp100-positive cells stained brownish-red with aminoethylcarbazole. (**A**) Spiked MMG-1 cells isolated from the peripheral blood of a healthy individual. The small light-brown spheroid is an immunomagnetic bead. (**B**) CTC detected in a patient with melanoma. Scar bar: 5 *μ*m.

To establish the optimal conditions for isolating CTC from peripheral blood, cultured melanoma cells were mixed with peripheral blood and isolated using immunomagnetic beads. Briefly, the melanoma cell lines 888mel, 928mel, or MMG1, which express HMW-MAA and MART-1/gp100, were spiked into healthy human peripheral blood samples at concentrations ranging from 1 × 10^2^ to 1 × 10^5^ cells per 5 ml blood. The mixtures were sequentially stained with mAbs specific for HMW-MAA- and CD45 and fractionated using immunomagnetic beads. A mixture of mAbs that recognise distinct and spatially distant epitopes on HMW-MAA was used to minimise false-negative results caused by differential expression of HMW-MAA epitopes, and to increase the detection level of the system (Kitago *et al*, 2009). To confirm that the isolated HMW-MAA^+^, CD45^−^ cells were melanoma cells, the cells were stained with MART-1- and gp100-specific mAbs and observed under a microscope (Figure 1A). Isolated HMW-MAA^+^, CD45^−^, MART-1/gp100^+^ cells comprised 1–24% of the melanoma cells initially mixed with the peripheral blood (Table 1). The three melanoma cell lines showed different recovery rates. The expression level of HMW-MAA was measured by reverse transcription PCR (RT–PCR) and flow cytometry, and that of MART-1/gp100 by immunostaining. Results showed no correlation between the recovery rates and the level of HMW-MAA or MART-1/gp100 (data not shown).

The possibility that mutant alleles may ‘drop-out’ during PCR, a known problem when using single cells, was assessed (Piyamongkol *et al*, 2003). The *BRAF* mutation was analysed in single HMW-MAA^+^, CD45^−^, MART-1/gp100^+^ cells isolated from blood mixed with 928mel cells, which harbour the heterozygous *BRAF*^*V600E*^ mutation (Lin *et al*, 2009). DNA was extracted from each cell and *BRAF* exon 15, which is the most common mutation site (Platz *et al*, 2008), was sequenced in each cell individually. The *BRAF*^*V600E*^ mutation was detected in 88.9% of the isolated 928mel cells (48 of 54 cells sequenced). The drop-out of mutant alleles during single-cell PCR is likely to be a relatively rare event; a previous study using a melanoma cell line showed that the phenomenon occurs at a frequency of only 2–14% (1–7 out of 50 single melanoma cells) (Lin *et al*, 2009). Thus, in this *in-vitro* model experiment, the *BRAF* mutation was successfully detected in individual melanoma cells isolated from peripheral blood.

### CTC separation from the peripheral blood of melanoma patients

Next, CTC were isolated from the peripheral blood of 11 patients with stage IIIC/IV melanoma (Table 2), all of which expressed HMW-MAA and MART-1/gp100, as detected by immunohistochemical analysis. Peripheral blood was incubated with the mAb cocktail and magnetic beads as described in the previous section. The HMW-MAA^+^, CD45^−^ cells were isolated and the cellular phenotype was confirmed by MART-1/gp100 expression. The HMW-MAA^+^, CD45^−^, MART-1/gp100^+^ cells were detected in all the patients. The isolated cells were larger and showed an abnormal morphology (Figure 1B). The yield of CTC from 5 ml of blood ranged from 1 to 20 (Table 2). Because MART-1/gp100^+^ cells could be identified microscopically, contamination by non-melanoma cells non-specifically bound to the immunomagnetic beads was unlikely. As controls, peripheral blood samples obtained from healthy individuals were included in every experiment. No HMW-MAA^+^, CD45^−^, MART-1/gp100^+^ CTC were isolated from the peripheral blood of >50 healthy donors.

### *BRAF* mutations in CTC from the peripheral blood of melanoma patients

**Figure 2:**
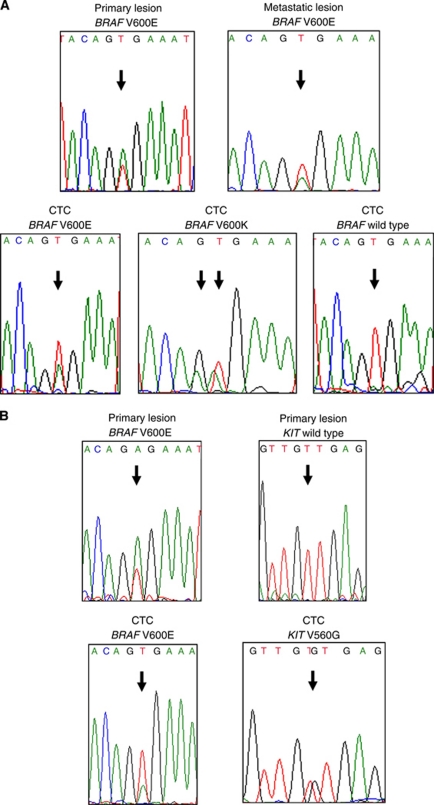
*BRAF* and *KIT* mutations detected in CTC isolated from melanoma patients. (**A**) Polyclonal *BRAF* mutations detected in CTC from patient No. 5. Primary and metastatic lesions showed *BRAF*^V600E^ (upper). Two CTC showed *BRAF*^V600E^ and *BRAF*^V600K^ mutations, respectively (lower left and middle), and another CTC possessed wild-type *BRAF* (lower right). (**B**) Concomitant detection of *BRAF*^V600E^ (lower left) and *KIT*^V560G^ (lower right) mutations in CTC obtained from patient No. 4. The primary lesion showed *BRAF*^V600E^ and wild-type *KIT* (upper).

CTC were captured individually. *BRAF* exon 15 was amplified from single CTC from 9 out of 11 melanoma patients and *BRAF* exon 15 sequences were obtained from 14 individual CTC (Table 3). Single CTC PCR occasionally failed; the success rate of PCR amplification from single CTC ranged from 20% to 100%. In three cases in which primary melanomas showed *BRAF*^V600E^ mutation, CTC shared a similar *BRAF* genotype with the primary tumours (Nos. 4, 5, and 6; Table 3). By contrast, lymph node metastases from patient No. 9 showed a *BRAF*^V600E^ mutation while two CTC showed wild-type *BRAF*. This result should be interpreted with caution because of the possibility of mutant allele drop-out in CTC; however, that phenomenon is likely to be relatively rare, as discussed earlier. Interestingly, CTC from patient No. 5 showed a *BRAF*^V600K^ mutation and a wild-type sequence in addition to the *BRAF*^V600E^ mutation (which was also identified in the primary tumour and lymph node metastasis; Figure 2A). This suggests clonal heterogeneity in terms of *BRAF* mutations during melanoma progression.

The *BRAF* genotype in metastases of patient Nos. 5, 6, and 9 was assessed further because these three cases showed a mismatched *BRAF* genotype between metastasis and CTC. Clusters of 30–50 melanoma cells were microdissected from different areas within metastatic lesions to detect minor populations of tumour cells, since conventional sequencing usually detects genetic mutations at a rate of >20% (Ellison *et al*, 2010). The microdissected lesions showed both *BRAF*^V600E^ and wild-type *BRAF* in Nos. 5 and 6, but not in No. 9 (Supplementary Table S2). Although the macrodissected lesions showed *BRAF*^V600E^ in No. 5 and wild type in No. 6, some microdissected samples showed different genotypes (wild type in No. 5 and *BRAF*^V600E^ in No. 6), suggesting that a substantial number of cells harboured different *BRAF* genotypes. Metastatic lesions may occasionally include heterogeneous tumour cells. The *BRAF*^V600K^ mutation was not detected in the metastasis of No. 5 nor was wild-type *BRAF* in that of No. 9. These data suggest that CTC with mismatched genotypes may be derived from minor populations within metastatic lesions.

### *KIT* mutations in CTC isolated from the peripheral blood of melanoma patients

The sequences within three exons (exons 11, 13, and 17) of *KIT*, which are reported to harbour mutations (Woodman and Davies, 2010), were also examined. The PCR amplification success rate ranged from 0% to 50% (Table 3). To optimise PCR conditions from single cells, several conditions were tested, including primers, enzymes, and buffers; however, the success rate did not improve sufficiently. *KIT* genotypes were analysed in only four patients (Table 3). Unlike *BRAF* genotypes, *KIT* genotypes were poorly matched between primary tumours, metastases, and CTC (Table 3). The primary tumours from two patients (Nos. 1 and 10) showed *KIT* mutations, but no *KIT* mutations were detectable in lymph node metastases and/or CTC. However, *KIT* mutations were detected in either metastases or CTC in patient Nos. 4 and 11, whereas the primary lesions showed wild-type *KIT*. It is interesting to note that patient No. 4 showed not only a *KIT*^V560G^ mutation in CTC, but also a *BRAF*^V600E^ mutation in the primary tumour, as well as in another CTC (Figure 2B); this is unusual because mutations in *BRAF* and *KIT* are thought to be mutually exclusive (Curtin *et al*, 2006; Carvajal *et al*, 2011). In addition, *KIT*^V560G^ has not been reported in melanomas, but is prevalent in gastrointestinal stromal tumours and mastocytosis (Tarn *et al*, 2005; Lanternier *et al*, 2008).

## Discussion

This study describes the purification of CTC from the peripheral blood of melanoma patients using immunomagnetic beads coated with HMW-MAA-specific antibodies followed by immunohistochemical laser dissection techniques. The *BRAF* and *KIT* genotypes of the isolated CTC were then analysed; the results showed that the genotypes of the CTC differed from those of the primary tumours and metastatic lesions. The method outlined in this study presents a new opportunity to characterise the genetic mutations expressed by spreading melanoma cells.

CTC represent a promising prognostic biomarker for melanoma recurrence or progression. Since subclinical, distant metastases occur during the early stages of melanoma development (Ossowski and Aguirre-Ghiso, 2010), detecting melanoma cells released from metastatic tumours may help to identify those melanoma patients at high risk of developing systemic metastatic disease. However, clinical detection of these cells is often extensively delayed after removal of the primary melanoma lesions.

CTC in the epithelial cancers are frequently isolated using immunomagnetic separation techniques incorporating antibodies against epithelial-specific antigens, such as EpCAM, BerEP4, and cytokeratins (Paterlini-Brechot and Benali, 2007; Krebs *et al*, 2010; Negin and Cohen, 2010; Riethdorf and Pantel, 2010). However, somewhat conflicting results have been published regarding the prognostic value of CTC, although it appears that increased CTC numbers are associated with more diffuse cancers, a higher risk of relapse, and a poor prognosis in breast, colon, and other epithelial cancers (Paterlini-Brechot and Benali, 2007; Krebs *et al*, 2010; Negin and Cohen, 2010; Riethdorf and Pantel, 2010). As epithelial-specific antibodies are of no use when attempting to isolate CTC from melanoma patients, CTC have been examined at the mRNA level using PCR-based methods. The presence of melanoma cell-specific mRNA seems to be associated with a poor outcome (Mellado *et al*, 1999; Palmieri *et al*, 1999; Pantel *et al*, 1999; Koyanagi *et al*, 2005); however, such approaches do not permit the genotypic or phenotypic characterisation of melanoma CTC. Therefore, cellular approaches to CTC detection were developed using immunomagnetic cell sorting or other methods (Ulmer *et al*, 2004; Galanzha *et al*, 2009; Kitago *et al*, 2009; De Giorgi *et al*, 2010; Suesskind *et al*, 2011). The HMW-MAA-specific mAbs have been used for the immunomagnetic separation of melanoma CTC (Ulmer *et al*, 2004; Kitago *et al*, 2009; Suesskind *et al*, 2011), but single step selection methods using HMW-MAA still result in blood cell contamination. The HMW-MAA^+^, CD45^−^, MART-1/gp100^+^ cells are morphologically different from blood cells and show atypical features, which can be used to confirm that isolated HMW-MAA^+^, CD45^−^, MART-1/gp100^+^ cells are, in fact, CTC. A potential drawback of this method is the low yield, which may be attributable to the immunomagnetic-negative selection of CD45^+^ cells, since a partial loss of CTC was observed during that process in patients with colon cancer (Ausch *et al*, 2007). Further technical improvements regarding the isolation procedure should aim at improving this aspect.

There are several powerful examples of the application of CTC genotyping to targeted cancer therapy. In lung cancer, molecular analysis of the epidermal growth factor receptor gene in CTC provides important information regarding therapeutic response and prognosis (Maheswaran *et al*, 2008). The human epidermal growth factor receptor-2 (*HER2*) gene is examined in CTC from breast cancer patients because HER2 antagonists are available for therapy. However, the HER2 status of the CTC is sometimes different from that of the primary tumours and metastatic lesions (Wulfing *et al*, 2006; Pestrin *et al*, 2009; Flores *et al*, 2010). Furthermore, a subset of patients with HER2-negative primary tumours developed HER2-positive CTC as the tumour progressed (Meng *et al*, 2004; Pestrin *et al*, 2009). Similarly, the results of the present study show that, although the *BRAF* genotype of the CTC was similar to that of the resected primary tumours, there were still some important mismatches. The *KIT* genotype showed the greatest variability between primary melanomas, lymph node metastases, and CTC. Since the features of CTC are not always concordant with those of other lesions, it is necessary to pay attention to the genotypes of both the primary/metastatic lesions and the CTC.

CTC from one patient (No. 5) showed three *BRAF* genotypes (*BRAF*^V600E^, *BRAF*^V600K^, and wild type). Analysis at the single cell level allowed further genetic dissection, as conventional sequencing techniques fail to detect minor mutations (Ellison *et al*, 2010). The polyclonal genotype implies that melanoma cells have heterogeneous features. It is noteworthy that oesophageal cancer cells disseminate to the bone marrow and lymph nodes at an early stage. Whole genome analysis at the single cell level showed that these disseminated tumour cells are genomically heterogeneous (Stoecklein *et al*, 2008). It is conceivable that, as in oesophageal cancer, heterogeneous melanoma cells may disseminate to various tissues at an early stage. *BRAF* genotyping from single melanoma cells showed heterogeneity within metastatic lesions and primary tumours (Lin *et al*, 2011). In the present study, *BRAF* heterogeneity was also detected within metastatic melanomas through the analysis of DNA isolated from dozens of tumour cells. Intralesional heterogeneity within metastatic lesions is likely to cause mismatched genotypes between CTC and macrodissected metastatic lesions. The CTC features may reflect the cellular state of active melanoma cells migrating from metastatic lesions.

*BRAF* heterogeneity has significant implications when using *RAF* kinase inhibitors because, in contrast to *BRAF*^V600E^ melanoma cells, *BRAF* wild-type cells are resistant to (and may even be stimulated by) *RAF* kinase inhibitors (Hatzivassiliou *et al*, 2010; Heidorn *et al*, 2010). Furthermore, most patients with *BRAF*^V600E^ mutations develop secondary resistance and show subsequent disease progression. Based on the heterogeneity of melanoma cells, secondary resistance may be attributable to *BRAF* heterogeneity. In this case, the number of wild-type *BRAF* CTC may alter after treatment with kinase inhibitors, and CTC analysis may help to identify the point at which cells ‘escape’, leading to resistance and relapse.

The CTC analysis also has potential benefits in terms of treating *KIT* mutant melanomas. Recent phase II clinical trials involving patients with metastatic melanoma harbouring *KIT* mutations reported significant clinical responses to imatinib in a subset of patients (Carvajal *et al*, 2011; Guo *et al*, 2011). However, tyrosine kinase inhibitors show little or no activity in melanoma cells harbouring wild-type *KIT* (Becker *et al*, 2007). In a particular case of melanoma with metastases in the lung and lymph nodes, imatinib treatment led to a rapid response in lung metastases harbouring the *KIT*^V559A^ mutation, but no response in lymph node metastases without the mutation (Terheyden *et al*, 2010). Thus, mutation screening is critical if we are to identify those patients that may benefit from the kinase-targeted therapy.

The present study has several limitations. First, the number of melanomas examined was small. Second, PCR amplification from single CTC occasionally failed; melanoma cells may include substances that inhibit PCR. Further improvement of single cell PCR methods may eliminate this limitation. Third, the metastatic potential of CTC is not clear. Furthermore, we do not have data to allow us to examine the relationship between the results of CTC genotyping and clinical outcome. However, CTC may provide real-time information regarding cancers at the whole-body level. More work is necessary to develop methods for the isolation and analysis of CTC, but there is no doubt that the information provided by CTC will improve the ability of clinicians to predict responses to molecular-targeted therapies.

## Figures and Tables

**Table 1:** Recovery of melanoma cells mixed with peripheral blood

	**Observed cell count**		
**No. of melanoma cells mixed in 5 ml of peripheral blood**	**888mel**	**928mel**	**MMG1**
10^5^	7600 (7.6%)	2420 (2.4%)	1920 (1.9%)
10^4^	1910 (19.1%)	260 (2.6%)	162 (1.6%)
10^3^	140 (14%)	20 (2%)	19 (1.9%)
10^2^	24 (24%)	1 (1%)	3 (3%)
0	0 (0%)	0 (0%)	0 (0%)

Results represent the average of two independent experiments.

**Table 2:** Clinical characteristics of the 11 melanoma patients

**No.**	**Sex**	**Age (years)**	**Histopathological type**	**AJCC stage**	**Metastatic sites**	**No. of CTC in 5 ml of PB**
1	M	54	Mucosal	IV	Lymph nodes, lung, brain, spinal cord	2–5
2	F	78	Mucosal	IV	Lymph nodes, lung, liver	2
3	M	67	Mucosal	IIIC	Lymph nodes	5
4	F	68	Mucosal	IV	Lymph nodes, lung	1–5
5	F	64	Non-CSD	IV	Lymph nodes, lung, liver, skin	1–20
6	M	61	Non-CSD	IV	Lymph nodes, lung, brain, skin	2–7
7	F	62	Non-CSD	IV	Lymph nodes, lung, peritoneum	1–3
8	M	73	Non-CSD	IV	Lymph nodes, digestive tract, pleura, abdominal cavity, skin	1
9	M	69	Unknown	IV	Lymph nodes, liver, digestive tract, bone, adrenal, skin	8
10	F	62	Acral	IIIC	Lymph nodes	6
11	M	84	Acral	IV	Lymph nodes, lung, brain	7

Abbreviations: CTC=circulating tumour cells; non-CSD=melanoma on non-chronic sun-damaged skin; PB=peripheral blood.

**Table 3:** Genotypes of *BRAF* and *KIT* in melanoma patients

	***BRAF* (exon 15)**				***KIT* (exons 11, 13, and 17)**			
	**Tissue**		**Single CTC**		**Tissue**		**Single CTC**	
**No.**	**Primary**	**Metastasis**	**No. of cells sequenced**	**Success rate of PCR**	**Primary**	**Metastasis**	**No. of cells sequenced**	**Success rate of PCR**
1	Wild type	Wild type	Wild type (1)	1/1	D820Y	Wild type	UR	0/3
2	Wild type	NA	Wild type (1)	1/1	Wild type	NA	UR	0/1
3	Wild type	NA	Wild type (1)	1/5	Wild type	NA	NE	NE
4	V600E	UR	V600E (1)	1/1	Wild type	UR	V560G (1)	1/2
5	V600E	V600E	V600E (1), V600K (1), wild type (1)	3/3	Wild type	Wild type	Wild type (1)	1/3
6	V600E	Wild type	V600E (3)	3/3	Wild type	Wild type	NE	NE
7	Wild type	Wild type	Wild type (1)	1/2	UR	Wild type	NE	NE
8	Wild type	Wild type	Wild type (1)	1/1	Wild type	Wild type	NE	NE
9	NA	V600E	Wild type (2)	2/4	NA	Wild type	NE	NE
10	Wild type	Wild type	NE	NE	N822Y	Wild type	Wild type (1)	1/6
11	Wild type	Wild type	NE	NE	Wild type	N822Y	Wild type (1)	1/2

Abbreviations: CTC=circulating tumour cells; NA=tissues not available; NE=not examined; UR=sequence unreadable due to PCR failure.

## References

[bib1] Allard WJ, Matera J, Miller MC, Repollet M, Connelly MC, Rao C, Tibbe AG, Uhr JW, Terstappen LW (2004) Tumor cells circulate in the peripheral blood of all major carcinomas but not in healthy subjects or patients with nonmalignant diseases. Clin Cancer Res 10: 6897–6904 15501967 10.1158/1078-0432.CCR-04-0378

[bib2] Ausch C, Dandachi N, Buxhofer-Ausch V, Balic M, Huber K, Bauernhofer T, Ogris E, Hinterberger W, Braun S, Schiessel R (2007) Immunomagnetic CD45 depletion does not improve cytokeratin 20 RT-PCR in colorectal cancer. Clin Chem Lab Med 45: 351–356 17378731 10.1515/CCLM.2007.059

[bib3] Becker JC, Brocker EB, Schadendorf D, Ugurel S (2007) Imatinib in melanoma: a selective treatment option based on KIT mutation status? J Clin Oncol 25: e9 17327598 10.1200/JCO.2006.08.9664

[bib4] Bustin SA, Benes V, Garson JA, Hellemans J, Huggett J, Kubista M, Mueller R, Nolan T, Pfaffl MW, Shipley GL, Vandesompele J, Wittwer CT (2009) The MIQE guidelines: minimum information for publication of quantitative real-time PCR experiments. Clin Chem 55: 611–622 19246619 10.1373/clinchem.2008.112797

[bib5] Buttner C, Henz BM, Welker P, Sepp NT, Grabbe J (1998) Identification of activating c-kit mutations in adult-, but not in childhood-onset indolent mastocytosis: a possible explanation for divergent clinical behavior. J Invest Dermatol 111: 1227–1231 9856847 10.1046/j.1523-1747.1998.00414.x

[bib6] Campoli MR, Chang CC, Kageshita T, Wang X, McCarthy JB, Ferrone S (2004) Human high molecular weight-melanoma-associated antigen (HMW-MAA): a melanoma cell surface chondroitin sulfate proteoglycan (MSCP) with biological and clinical significance. Crit Rev Immunol 24: 267–296 15588226 10.1615/critrevimmunol.v24.i4.40

[bib7] Carvajal RD, Antonescu CR, Wolchok JD, Chapman PB, Roman RA, Teitcher J, Panageas KS, Busam KJ, Chmielowski B, Lutzky J, Pavlick AC, Fusco A, Cane L, Takebe N, Vemula S, Bouvier N, Bastian BC, Schwartz GK (2011) KIT as a therapeutic target in metastatic melanoma. JAMA 305: 2327–2334 21642685 10.1001/jama.2011.746 PMC3986039

[bib8] Chapman PB, Hauschild A, Robert C, Haanen JB, Ascierto P, Larkin J, Dummer R, Garbe C, Testori A, Maio M, Hogg D, Lorigan P, Lebbe C, Jouary T, Schadendorf D, Ribas A, O'Day SJ, Sosman JA, Kirkwood JM, Eggermont AM, Dreno B, Nolop K, Li J, Nelson B, Hou J, Lee RJ, Flaherty KT, McArthur AG (2011) Improved survival with vemurafenib in melanoma with BRAF V600E mutation. N Engl J Med 364: 2507–2516 21639808 10.1056/NEJMoa1103782 PMC3549296

[bib9] Curtin JA, Busam K, Pinkel D, Bastian BC (2006) Somatic activation of KIT in distinct subtypes of melanoma. J Clin Oncol 24: 4340–4346 16908931 10.1200/JCO.2006.06.2984

[bib10] Davies H, Bignell GR, Cox C, Stephens P, Edkins S, Clegg S, Teague J, Woffendin H, Garnett MJ, Bottomley W, Davis N, Dicks E, Ewing R, Floyd Y, Gray K, Hall S, Hawes R, Hughes J, Kosmidou V, Menzies A, Mould C, Parker A, Stevens C, Watt S, Hooper S, Wilson R, Jayatilake H, Gusterson BA, Cooper C, Shipley J, Hargrave D, Pritchard-Jones K, Maitland N, Chenevix-Trench G, Riggins GJ, Bigner DD, Palmieri G, Cossu A, Flanagan A, Nicholson A, Ho JW, Leung SY, Yuen ST, Weber BL, Seigler HF, Darrow TL, Paterson H, Marais R, Marshall CJ, Wooster R, Stratton MR, Futreal PA (2002) Mutations of the BRAF gene in human cancer. Nature 417: 949–954 12068308 10.1038/nature00766

[bib11] De Giorgi V, Pinzani P, Salvianti F, Panelos J, Paglierani M, Janowska A, Grazzini M, Wechsler J, Orlando C, Santucci M, Lotti T, Pazzagli M, Massi D (2010) Application of a filtration- and isolation-by-size technique for the detection of circulating tumor cells in cutaneous melanoma. J Invest Dermatol 130: 2440–2447 20535130 10.1038/jid.2010.141

[bib12] Ellison G, Donald E, McWalter G, Knight L, Fletcher L, Sherwood J, Cantarini M, Orr M, Speake G (2010) A comparison of ARMS and DNA sequencing for mutation analysis in clinical biopsy samples. J Exp Clin Cancer Res 29: 132 20925915 10.1186/1756-9966-29-132 PMC2988723

[bib13] Flaherty KT, Puzanov I, Kim KB, Ribas A, McArthur GA, Sosman JA, O'Dwyer PJ, Lee RJ, Grippo JF, Nolop K, Chapman PB (2010) Inhibition of mutated, activated BRAF in metastatic melanoma. N Engl J Med 363: 809–819 20818844 10.1056/NEJMoa1002011 PMC3724529

[bib14] Flores LM, Kindelberger DW, Ligon AH, Capelletti M, Fiorentino M, Loda M, Cibas ES, Janne PA, Krop IE (2010) Improving the yield of circulating tumour cells facilitates molecular characterisation and recognition of discordant HER2 amplification in breast cancer. Br J Cancer 102: 1495–1502 20461092 10.1038/sj.bjc.6605676 PMC2869174

[bib15] Galanzha EI, Shashkov EV, Spring PM, Suen JY, Zharov VP (2009) *In vivo*, noninvasive, label-free detection and eradication of circulating metastatic melanoma cells using two-color photoacoustic flow cytometry with a diode laser. Cancer Res 69: 7926–7934 19826056 10.1158/0008-5472.CAN-08-4900 PMC2828368

[bib16] Guo J, Si L, Kong Y, Flaherty KT, Xu X, Zhu Y, Corless CL, Li L, Li H, Sheng X, Cui C, Chi Z, Li S, Han M, Mao L, Lin X, Du N, Zhang X, Li J, Wang B, Qin S (2011) Phase II, open-label, single-arm trial of imatinib mesylate in patients with metastatic melanoma harboring c-Kit mutation or amplification. J Clin Oncol 29: 2904–2909 21690468 10.1200/JCO.2010.33.9275

[bib17] Hatzivassiliou G, Song K, Yen I, Brandhuber BJ, Anderson DJ, Alvarado R, Ludlam MJ, Stokoe D, Gloor SL, Vigers G, Morales T, Aliagas I, Liu B, Sideris S, Hoeflich KP, Jaiswal BS, Seshagiri S, Koeppen H, Belvin M, Friedman LS, Malek S (2010) RAF inhibitors prime wild-type RAF to activate the MAPK pathway and enhance growth. Nature 464: 431–435 20130576 10.1038/nature08833

[bib18] Heidorn SJ, Milagre C, Whittaker S, Nourry A, Niculescu-Duvas I, Dhomen N, Hussain J, Reis-Filho JS, Springer CJ, Pritchard C, Marais R (2010) Kinase-dead BRAF and oncogenic RAS cooperate to drive tumor progression through CRAF. Cell 140: 209–221 20141835 10.1016/j.cell.2009.12.040 PMC2872605

[bib19] Hofmann UB, Kauczok-Vetter CS, Houben R, Becker JC (2009) Overexpression of the KIT/SCF in uveal melanoma does not translate into clinical efficacy of imatinib mesylate. Clin Cancer Res 15: 324–329 19118061 10.1158/1078-0432.CCR-08-2243

[bib20] Kitago M, Koyanagi K, Nakamura T, Goto Y, Faries M, O'Day SJ, Morton DL, Ferrone S, Hoon DS (2009) mRNA expression and BRAF mutation in circulating melanoma cells isolated from peripheral blood with high molecular weight melanoma-associated antigen-specific monoclonal antibody beads. Clin Chem 55: 757–764 19233913 10.1373/clinchem.2008.116467 PMC2760934

[bib21] Koyanagi K, Kuo C, Nakagawa T, Mori T, Ueno H, Lorico Jr AR, Wang HJ, Hseuh E, O'Day SJ, Hoon DS (2005) Multimarker quantitative real-time PCR detection of circulating melanoma cells in peripheral blood: relation to disease stage in melanoma patients. Clin Chem 51: 981–988 15817820 10.1373/clinchem.2004.045096 PMC2856477

[bib22] Krebs MG, Hou JM, Ward TH, Blackhall FH, Dive C (2010) Circulating tumour cells: their utility in cancer management and predicting outcomes. Ther Adv Med Oncol 2: 351–365 21789147 10.1177/1758834010378414 PMC3126032

[bib23] Lanternier F, Cohen-Akenine A, Palmerini F, Feger F, Yang Y, Zermati Y, Barete S, Sans B, Baude C, Ghez D, Suarez F, Delarue R, Casassus P, Bodemer C, Catteau A, Soppelsa F, Hanssens K, Arock M, Sobol H, Fraitag S, Canioni D, Moussy A, Launay JM, Dubreuil P, Hermine O, Lortholary O (2008) Phenotypic and genotypic characteristics of mastocytosis according to the age of onset. PLoS One 3: e1906 18404201 10.1371/journal.pone.0001906 PMC2292130

[bib24] Lin J, Goto Y, Murata H, Sakaizawa K, Uchiyama A, Saida T, Takata M (2011) Polyclonality of BRAF mutations in primary melanoma and the selection of mutant alleles during progression. Br J Cancer 104: 464–468 21224857 10.1038/sj.bjc.6606072 PMC3049568

[bib25] Lin J, Takata M, Murata H, Goto Y, Kido K, Ferrone S, Saida T (2009) Polyclonality of BRAF mutations in acquired melanocytic nevi. J Natl Cancer Inst 101: 1423–1427 19752400 10.1093/jnci/djp309 PMC2765260

[bib26] Maheswaran S, Sequist LV, Nagrath S, Ulkus L, Brannigan B, Collura CV, Inserra E, Diederichs S, Iafrate AJ, Bell DW, Digumarthy S, Muzikansky A, Irimia D, Settleman J, Tompkins RG, Lynch TJ, Toner M, Haber DA (2008) Detection of mutations in EGFR in circulating lung-cancer cells. N Engl J Med 359: 366–377 18596266 10.1056/NEJMoa0800668 PMC3551471

[bib27] Mellado B, Gutierrez L, Castel T, Colomer D, Fontanillas M, Castro J, Estape J (1999) Prognostic significance of the detection of circulating malignant cells by reverse transcriptase-polymerase chain reaction in long-term clinically disease-free melanoma patients. Clin Cancer Res 5: 1843–1848 10430090

[bib28] Meng S, Tripathy D, Shete S, Ashfaq R, Haley B, Perkins S, Beitsch P, Khan A, Euhus D, Osborne C, Frenkel E, Hoover S, Leitch M, Clifford E, Vitetta E, Morrison L, Herlyn D, Terstappen LW, Fleming T, Fehm T, Tucker T, Lane N, Wang J, Uhr J (2004) HER-2 gene amplification can be acquired as breast cancer progresses. Proc Natl Acad Sci USA 101: 9393–9398 15194824 10.1073/pnas.0402993101 PMC438987

[bib29] Mocellin S, Keilholz U, Rossi CR, Nitti D (2006) Circulating tumor cells: the ‘leukemic phase’ of solid cancers. Trends Mol Med 12: 130–139 16488189 10.1016/j.molmed.2006.01.006

[bib30] Negin BP, Cohen SJ (2010) Circulating tumor cells in colorectal cancer: past, present, and future challenges. Curr Treat Options Oncol 11: 1–13 20143276 10.1007/s11864-010-0115-3

[bib31] Ossowski L, Aguirre-Ghiso JA (2010) Dormancy of metastatic melanoma. Pigment Cell Melanoma Res 23: 41–56 19843243 10.1111/j.1755-148X.2009.00647.x PMC2821074

[bib32] Palmieri G, Strazzullo M, Ascierto PA, Satriano SM, Daponte A, Castello G (1999) Polymerase chain reaction-based detection of circulating melanoma cells as an effective marker of tumor progression. Melanoma Cooperative Group. J Clin Oncol 17: 304–311 10458247 10.1200/JCO.1999.17.1.304

[bib33] Pantel K, Cote RJ, Fodstad O (1999) Detection and clinical importance of micrometastatic disease. J Natl Cancer Inst 91: 1113–1124 10393719 10.1093/jnci/91.13.1113

[bib34] Paterlini-Brechot P, Benali NL (2007) Circulating tumor cells (CTC) detection: clinical impact and future directions. Cancer Lett 253: 180–204 17314005 10.1016/j.canlet.2006.12.014

[bib35] Pestrin M, Bessi S, Galardi F, Truglia M, Biggeri A, Biagioni C, Cappadona S, Biganzoli L, Giannini A, Di Leo A (2009) Correlation of HER2 status between primary tumors and corresponding circulating tumor cells in advanced breast cancer patients. Breast Cancer Res Treat 118: 523–530 19597704 10.1007/s10549-009-0461-7

[bib36] Piyamongkol W, Bermudez MG, Harper JC, Wells D (2003) Detailed investigation of factors influencing amplification efficiency and allele drop-out in single cell PCR: implications for preimplantation genetic diagnosis. Mol Hum Reprod 9: 411–420 12802048 10.1093/molehr/gag051

[bib37] Platz A, Egyhazi S, Ringborg U, Hansson J (2008) Human cutaneous melanoma; a review of NRAS and BRAF mutation frequencies in relation to histogenetic subclass and body site. Mol Oncol 1: 395–405 19383313 10.1016/j.molonc.2007.12.003 PMC5543839

[bib38] Riethdorf S, Pantel K (2010) Advancing personalized cancer therapy by detection and characterization of circulating carcinoma cells. Ann NY Acad Sci 1210: 66–77 20973800 10.1111/j.1749-6632.2010.05779.x

[bib39] Romano E, Schwartz GK, Chapman PB, Wolchock JD, Carvajal RD (2011) Treatment implications of the emerging molecular classification system for melanoma. Lancet Oncol 12: 913–922 21349766 10.1016/S1470-2045(10)70274-6

[bib40] Stoecklein NH, Hosch SB, Bezler M, Stern F, Hartmann CH, Vay C, Siegmund A, Scheunemann P, Schurr P, Knoefel WT, Verde PE, Reichelt U, Erbersdobler A, Grau R, Ullrich A, Izbicki JR, Klein CA (2008) Direct genetic analysis of single disseminated cancer cells for prediction of outcome and therapy selection in esophageal cancer. Cancer Cell 13: 441–453 18455127 10.1016/j.ccr.2008.04.005

[bib41] Suesskind D, Ulmer A, Schiebel U, Fierlbeck G, Spitzer B, Spitzer MS, Bartz-Schmidt KU, Grisanti S (2011) Circulating melanoma cells in peripheral blood of patients with uveal melanoma before and after different therapies and association with prognostic parameters: a pilot study. Acta Ophthalmol 89: 17–24 21272286 10.1111/j.1755-3768.2009.01617.x

[bib42] Tarn C, Merkel E, Canutescu AA, Shen W, Skorobogatko Y, Heslin MJ, Eisenberg B, Birbe R, Patchefsky A, Dunbrack R, Arnoletti JP, von Mehren M, Godwin AK (2005) Analysis of KIT mutations in sporadic and familial gastrointestinal stromal tumors: therapeutic implications through protein modeling. Clin Cancer Res 11: 3668–3677 15897563 10.1158/1078-0432.CCR-04-2515

[bib43] Terheyden P, Houben R, Pajouh P, Thorns C, Zillikens D, Becker JC (2010) Response to imatinib mesylate depends on the presence of the V559A-mutated KIT oncogene. J Invest Dermatol 130: 314–316 19812602 10.1038/jid.2009.197

[bib44] Ulmer A, Schmidt-Kittler O, Fischer J, Ellwanger U, Rassner G, Riethmuller G, Fierlbeck G, Klein CA (2004) Immunomagnetic enrichment, genomic characterization, and prognostic impact of circulating melanoma cells. Clin Cancer Res 10: 531–537 14760074 10.1158/1078-0432.ccr-0424-03

[bib45] Woodman SE, Davies MA (2010) Targeting KIT in melanoma: a paradigm of molecular medicine and targeted therapeutics. Biochem Pharmacol 80: 568–574 20457136 10.1016/j.bcp.2010.04.032 PMC3935736

[bib46] Wulfing P, Borchard J, Buerger H, Heidl S, Zanker KS, Kiesel L, Brandt B (2006) HER2-positive circulating tumor cells indicate poor clinical outcome in stage I to III breast cancer patients. Clin Cancer Res 12: 1715–1720 16551854 10.1158/1078-0432.CCR-05-2087

