# Supplementary material for: Mutation analysis of BRAF and KIT in circulating melanoma cells at the single cell level
Source: Br J Cancer. 2012 Jan 26;106(5):939–46. doi: 10.1038/bjc.2012.12 (PMC3305957; doi:10.1038/bjc.2012.12)
Supplement: Supplementary Information [file bjc201212x1.doc]

**SUPPLEMENTAL EXPERIMENTAL PROCEDURES**

**Melanoma cell lines and reagents**

The melanoma cell lines 888mel and 928mel (obtained from Prof. Y. Kawakami; Keio University School of Medicine, Tokyo, Japan), and MMG1 (obtained from Prof. A. Yamamoto; Saitama Medical University, Hidaka, Japan) were cultured in RPMI-1640 medium, supplemented with 5% heat inactivated fetal bovine serum and 1% penicillin-streptomycin at 37°C in a humidified 5% CO_2_-enriched atmosphere. Cells were fed twice per week and passaged by treatment with 0.05% trypsin in ethylenediamine tetra-acetic acid.

**Patients**

All the melanoma patients enrolled in this study provided written, informed consent for the use of blood samples and resected tissues. The study was approved by the Ethical Committee of Shinshu University School of Medicine and carried out according to the guidelines set out by the Institutional Review Board. Clinical stages were defined according to the 2010 American Joint Committee on Cancer (AJCC) guidelines. Eleven patients, all with AJCC stage IIIC or IV melanoma, were studied. Immunohistochemistry showed the primary and metastatic melanoma lesions to be HMW-MAA^+^ and MART-1/gp100^+^.

**Monoclonal antibodies**

mAbs 763.74, VF1-TP41.2, and VT80.12, which recognise distinct and spatially separate epitopes on HMW-MAA, were developed and characterised as described (Campo*li et* al, 2004; Giacomi*ni et* al, 1985). mAbs were purified from ascites by sequential precipitation with caprylic acid and ammonium sulfate (Tempo*ni et* al, 1989). The purity and activity of the mAb preparations were assessed by SDS-PAGE and by binding to melanoma cells. MART-1-specific mouse mAb (clone M2-7C10) and human gp100-specific mouse mAb (clone HMB45) were purchased from Convance (Emeryville, CA, USA) and Dako, respectively.

**SUPPLEMENTAL REFERENCES**

Giacomini P, Natali P, Ferrone S (1985) Analysis of the interaction between a human high molecular weight melanoma-associated antigen and the monoclonal antibodies to three distinct antigenic determinants. *J Immunol* **135:** 696-702

Temponi M, Kageshita T, Perosa F, Ono R, Okada H, Ferrone S (1989) Purification of murine IgG monoclonal antibodies by precipitation with caprylic acid: comparison with other methods of purification. *Hybridoma* **8:** 85-95
