# Supplementary material for: Mutation analysis of BRAF and KIT in circulating melanoma cells at the single cell level
Source: Br J Cancer. 2012 Jan 26;106(5):939–46. doi: 10.1038/bjc.2012.12 (PMC3305957; doi:10.1038/bjc.2012.12)
Supplement: Supplementary Table S1 [file bjc201212x2.doc]

**Table S1.** Primers and cycling conditions used for PCR

|  | Exon | Outer primer | Inner primer | Cycling conditions for 1^st^ PCR | Cycling conditions for 2^nd^ PCR |
| --- | --- | --- | --- | --- | --- |
| *KIT* | 11 | Forward: 5’-GTTGGGAGGTGGGGTCAGTTT-3’  Reverse: 5’-GTTGGGAGGTGGGGTCAGTTT-3 | Forward: 5’-TGTTCTCTCTCCAGAGTGCTCTAA-3’  Reverse: 5’-AAACAAAGGAAGCCACTGGA-3’ | 25 cycles:  95°C for 30 seconds,  58°C for 30 seconds,  72°C for 30 seconds | 35 cycles:  95°C for 30 seconds,  58°C or 30 seconds,  72°C for 30 seconds |
|  | 13 | Forward: 5’-TTTCGGGAAGGTTGTTGAG-3’  Reverse: 5’-TACCCCATAATGATAAAAT-3’ | Forward: 5’-CATCAGTTTGCCAGTTGTGC-3’  Reverse: 5’-AGCAAGAGAGAACAACAGTCT-3’ |  |  |
|  |  |  |  |  |  |
|  | 17 | Forward: 5’-CAACCTAATAGTGTATTCACA-3’  Reverse: 5’- CTCACGTTTCCTTTAACCACATAA-3’ | Forward: 5’-GAGACTTGGCAGCCAGAAAT-3’  Reverse: 5’- CGTTTCCTTTAACCACATAA-3’ | 35 cycles:  94°C for 1 minute,  57°C for 1 minute,  72°C for 30 seconds | 25 cycles:  94°C for 1 minute,  57°C for 1 minute,  72°C for 30 seconds |
|  |  |  |  |  |  |
| *BRAF* | 15 | Forward: 5’-CATAATGCTTGCTCTGATAGG-3’  Reverse: 5’-GGCCAAAAATTTAATCAGTGGA-3’ | Forward: 5’- CATAATGCTTGCTCTGATAGG-3’  Reverse: 5’-TAGCCTCAATTCTTACCATC-3’ | 22 cycles:  95°C for 25 seconds,  55°C for 25 seconds,  72°C for 30 seconds | 35 cycles:  95°C for 20 seconds,  55°C for 20 seconds,  72°C for 30 seconds |
