# Supplementary material for: Mutation analysis of BRAF and KIT in circulating melanoma cells at the single cell level
Source: Br J Cancer. 2012 Jan 26;106(5):939–46. doi: 10.1038/bjc.2012.12 (PMC3305957; doi:10.1038/bjc.2012.12)
Supplement: Supplementary Table S2 [file bjc201212x3.doc]

**Table S2.** Heterogenous *BRAF* genotype in metastatic lesions

|  |  |  |  |  |  |
| --- | --- | --- | --- | --- | --- |
|  |  | Metastasis | |  | Single CTC |
| No. |  | Macrodissected lesions | Microdissected lesions (No. of pieces sequenced) |  | (No. of cells sequenced) |
| 5 |  | V600E | V600E (4), wild (4) |  | V600E (1), V600K (1), wild (1) |
| 6 |  | Wild type | V600E (1), wild (2) |  | V600E (3) |
| 9 |  | V600E | V600E (7) |  | Wild type (2) |
|  |  |  |  |  |  |
